# Heart Transplantation in Mustard Patients Bridged With Continuous Flow Systemic Ventricular Assist Device - A Case Report and Review of Literature

**DOI:** 10.3389/fcvm.2021.651496

**Published:** 2021-04-20

**Authors:** Rody G. Bou Chaaya, Joel W. Simon, Mark Turrentine, Jeremy L. Herrmann, William Aaron Kay, Maya Guglin, Kashif Saleem, Roopa A. Rao

**Affiliations:** ^1^Department of Medicine, Indiana University School of Medicine, Indianapolis, IN, United States; ^2^Krannert Institute of Cardiology, Indiana University School of Medicine, Indianapolis, IN, United States; ^3^Division of Pediatric Cardiothoracic Surgery, Riley Hospital for Children, Indianapolis, IN, United States; ^4^Division of Cardiothoracic Surgery, Indiana University School of Medicine, Indianapolis, IN, United States

**Keywords:** ventricular assist device, mustard procedure, heart transplantation, pulmonary hypertension, d-TGA, transposition of the great arteries, pulmonary hypertension reversal

## Abstract

Thirty four-year-old male with history of D-transposition of the great arteries (D-TGA) who underwent Mustard operation at 14 months of age presented in cardiogenic shock secondary to severe systemic right ventricular failure. Catheterization revealed significantly increased pulmonary pressures. Due to the patient's inotrope dependence and prohibitive pulmonary hypertension, he underwent implantation of a Heart Ware HVAD® for systemic RV support. Within 4 months of continuous flow ventricular assist device (VAD) implantation complete normalization of pulmonary vascular resistance (PVR) was achieved. He ultimately underwent orthotopic heart transplantation with favorable outcomes. This is the second report of complete normalization of PVR following VAD implantation into a systemic RV in <4 months. We conducted a thorough literature review to identify Mustard patients that received systemic RV VAD as a bridge to a successful heart transplantation. In this article, we summarize the outcomes and focus on pulmonary hypertension reversibility following VAD implant.

## Introduction

Systemic right ventricular (RV) failure is a well-known sequel in the decades following atrial switch for D-transposition of the great arteries (D-TGA). As patients who underwent these procedures are now entering adulthood, at times reaching their 4th and even 5th decade of life, the disease burden can only be expected to grow. Cardiac transplantation is a well-accepted therapy for systemic RV failure, but the complication of secondary pulmonary hypertension may often be prohibitive. We herein report a case of severe systemic RV failure and pulmonary hypertension, with subsequent complete normalization of pulmonary vascular resistance (PVR) following continuous flow ventricular assist device (VAD) implantation.

### Case Presentation

**Figure 1:**
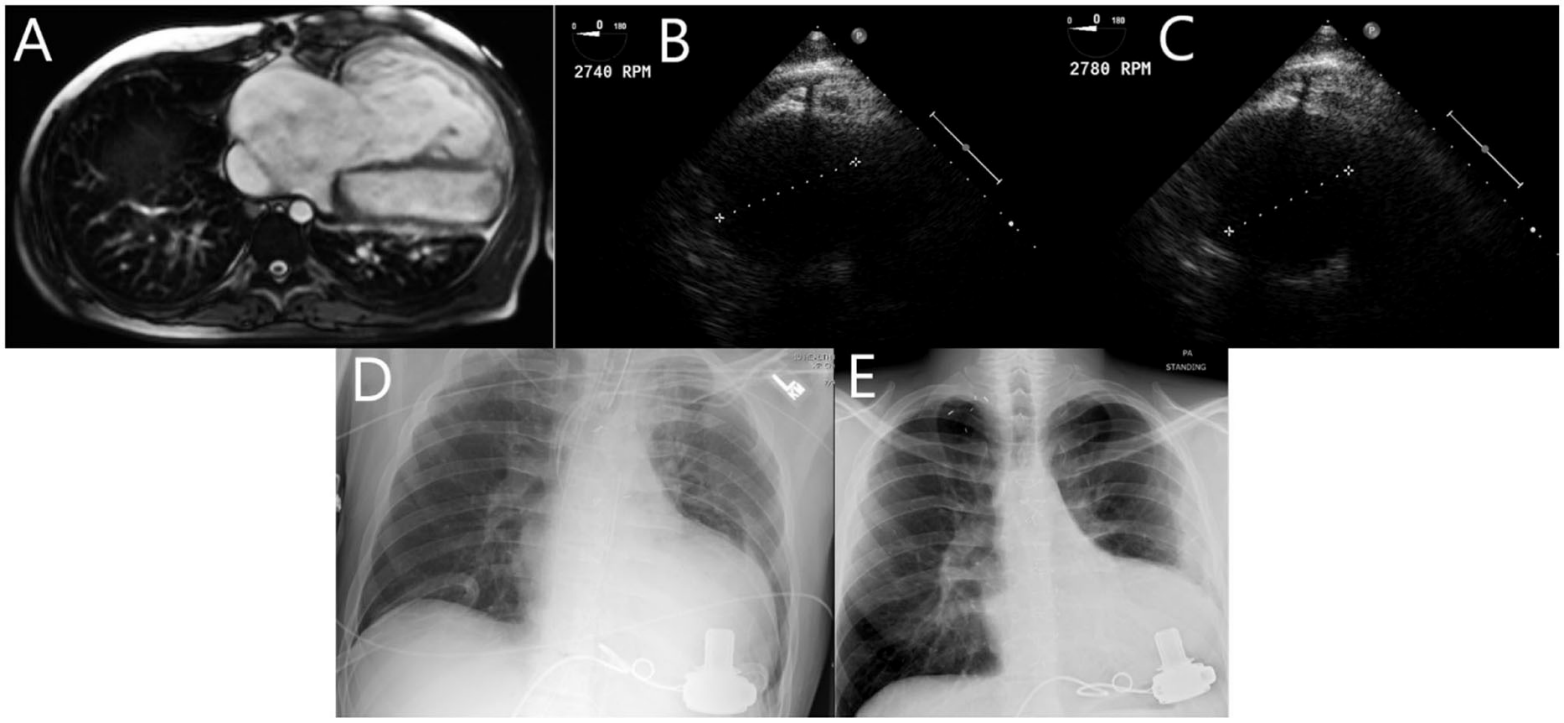
**(A)**: Cardiac MRI showing severely dilated and hypertrophied RV with mildly diminished systolic function. **(B, C)**: TEE monitoring showing RV chamber size decrease from 6.2 cm at low VAD speed (2,740 RPM) to 5.3 cm at high VAD speed (2,780 RPM) **(D, E)**: Chest x-ray showing significant decrease in RV size between post-operative day 1 and 10 months post-operatively.

Our patient is a 34-year-old male with history of D-TGA who underwent Mustard operation at 14 months of age. Surveillance monitoring showed stable pulmonary pressures, but slowly decreasing RV ejection fraction (RVEF 55% in 2007 to 42% in 2013) and increasing RV end diastolic volume (RVEDV 221 ml in 2007 to 423 ml in 2013) (Table 1). Cardiac magnetic resonance imaging (MRI) in 2013 showed severely dilated and hypertrophied RV (Figure 1A). In June 2014, the patient was admitted with worsening heart failure. He was hypotensive (blood pressure: 86/57 mmHg), lactate was 2.8 mmol/L. He also had evidence of multiorgan failure requiring inotropic support. Catheterization revealed significantly increased mean pulmonary artery pressure (MPAP) at 52 mmHg and PVR at 5.5 wood units (WU), as well as decreased cardiac index (CI) at 1.7 L/min/m^2^. Due to the patient's inotrope dependence and prohibitive elevation in PVR (> 4 WU), he underwent implantation of a Heart Ware HVAD® for systemic RV support. He tolerated the procedure very well. VAD flows were titrated with echocardiographic monitoring, RV chamber size decreased from 6.2 cm at low VAD speed [2,740 revolutions per minute (rpms)] to 5.3 cm at high VAD speed (2,780 rpms) (Figures 1B,C). He was successfully discharged on post-operative day 22.

**Table 1A:** Cardiac catheterization data over the 3 years before and 2 years after VAD placement.

**Date**	**MPAP**	**PVR**	**RVEDP**	**PCWP**	**CI**
	**(mmHg)**	**(Wood's Units)**	**(mmHg)**	**(mmHg)**	**(L/min/m^**2**^)**
**5/24/2012**	34	2.5	14	22	2.4
**10/29/2013**	35	3	20	23	2.5
**7/2/2014**	52	5.5	21	35	1.7
**11/3/2014**	**27**	**1.5**	**NR**	**18**	**3**
**5/27/2016**	**14**	**1.5**	**NR**	**7**	**2.2**

*Bolded text represents data after VAD. MPAP, mean pulmonary artery pressure; PVR, pulmonary vacular resistance; RVEDP, right ventricular end diastalic pressure; PCWP, pulmonary capillary wedge pressure; Cl, cardiac index; NR, not recorded*.

**Table 1B:** Cardiac MRI data for patient over several years prior to VAD implantation.

**Date**	**RVEF**	**RVEDV**	**CO**
	**(%)**	**(ml)**	**(L/min)**
**5/8/2007**	55.5	221.1	4.6
**2/9/2010**	43.2	280.5	5.3
**12/7/2011**	48	325.4	5.8
**10/2/2013**	41.9	423.6	3.9

*RVEF, right ventricular ejection fraction; RVEDV, right ventricular end diastalic volume; CQ, cardiac output*.

B-type natriuretic peptide decreased markedly post VAD implantation. Cardiac MRI could not be repeated due to the presence of the VAD, but chest x-ray showed significant decrease in RV size between post-operative day 1 and 10 months postoperatively (Figures 1D,E). The patient has not had any unplanned hospital readmissions and was able to walk more than one mile daily without symptoms after that. Repeat catheterization in 4 months showed complete normalization of PVR at 1.5 WU, and a CI of 3 L/min/m2. MPAP was 27 mmHg at that time, it continued to decrease to 14 mmHg in 2016.

Because of normalization of pulmonary resistance, he was ultimately listed for cardiac transplantation (UNOS status 1B). A suitable donor was identified in February 2017. He underwent VAD extraction and orthotopic heart transplantation (OHT) at that time. Surgical course was complicated by significant blood loss, requiring multiple units of blood products. Otherwise, he tolerated the transplantation very well. Immune suppression was induced with anti-thymocyte globulin, then transitioned to a regimen consisting of tacrolimus, mycophenolate mofetil, and prednisone. Symptoms continued to improve at 3 years post-transplant (New York Heart Association class I) and the patient's quality of life improved given that he is more active. Myocardial biopsies showed no signs of rejection and prednisone was weaned successfully.

## Discussion

The Mustard procedure is one of the two atrial switch operations initially performed in the 1960s to correct D-TGA. During this operation, a synthetic material is used to construct a two-way baffle. This generates a discordant atrioventricular connection on the existing discordant ventriculoarterial connection (1). The Mustard procedure has been widely replaced by the arterial switch operation in the late 1980s (1). Mustard patients survive to adulthood and now can make it to the 4th and 5th decade of life. However, long-term sequelae of Mustard physiology include baffle obstruction, baffle leaks, sinus node dysfunction, tachyarrhythmias and failure of the systemic RV. In one study of long-term outcomes, 61% of Mustard patients had moderate-to-severe systemic RV dysfunction 25 years after the surgery, 33% had mild dysfunction and only 6 % maintained normal ventricular function (2). Failing systemic RV can sometimes be supported by VAD, as either destination therapy or bridge to transplantation. However, widespread utilization is limited due to difficult surgical technique and other challenges. In the largest study of outcomes of VAD in adults with congenital heart disease (ACHD), 126 patients had VAD implants; of these, only 45 had a systemic morphologic RV (including both congenitally corrected transposition and atrial switch patients). Data from this study supported that survival after VAD was similar for both ACHD and non-ACHD patients (3).

Heart transplantation is the definitive long-term option for a failing systemic RV. There are few case-reports that describe VAD implantation for RV failure in patients with prior history of atrial switch as a bridge to a successful heart transplantation. Therefore, we conducted a thorough literature review using PubMed, Ovid/Medline, Cochrane Library to identify Mustard patients that received systemic RV VAD as a bridge to a successful heart transplantation between 1988 and 2020. A total of 13 published cases (4–12) were identified. Maly et al. (4) published a case series describing 5 adult Mustard patients with TGA who were supported by the Heart Mate II. Three patients had subsequent heart transplants, but 2 patients died of early and of chronic non-systemic ventricular failure, respectively, after VAD implant. Michel et al. (7) published another case series of 7 patients with d-TGA and CCTGA. One patient died perioperatively of recurrent VAD thrombosis; 2 patients had heart transplants and 4 patients remained on VAD support. The results are summarized in Table 2.

**Table 2:** Summary of articles describing systemic CF-VAD implantation for RV failure in patients with prior history of atrial switch as a bridge to a successful heart transplantation.

**Author, publication year & reference number**	**Patient profile (age in years at VAD implantation, sex)**	**Device type**	**Time on device**	**Total time on heart transplant waitlist**	**Indication for VAD**	**Pulmonary data**	**Pulmonary Hypertension reversal + duration**	**Mean PAP before VAD (mmHg)**	**Mean PAP after VAD (mmHg)**	**PVR before VAD (WU)**	**PVR after VAD (WU)**	**Stroke**	**Thrombosis**	**Drive line infection**	**OHT outcomes**
Maly J et al., 2015 (4)	29, male	Heart Mate II	11 months	11 months	Cardiogenic shock	Yes	N/A	14	NR	1.2	**NR**	**No**	**No**	**Yes**	**Alive, post-operative f/u**
	31, male		7 months	7 months	BTT	Yes	N/A	32	NR	1.7	**NR**	**No**	**No**	**Yes**	**Alive, post-operative f/u**
	33, male		12 months	12 months	BTT	Yes	N/A	37	NR	2.7	**NR**	**No**	**No**	**No**	**Alive, post-operative f/u**
Sugiura et al., 2018 (5)	34, male	Heart Mate II	72 months	84 months	BTT	No	–	–	–	–	**-**	**No**	**No**	**Yes**	**Alive, unspecified f/u**
Stewart AS et al., 2002 (6)	15, female	Heart Mate I	3 months	3 months	Cardiogenic shock	Yes	–	NR	–	8	**-**	**No**	**No**	**No**	**Alive, 3-year f/u**
Michel E et al., 2018 (7)	50, male	Heart Mate II	7.5 months	NR	BTT	Yes	No - 7.5 months	59	45	3.9	**3.8**	**No**	**No**	**No**	**Alive, post-operative f/u**
	40, male	HVAD	15 months		Severe pHTN then BTT	Yes	No - 15 months	76	30	8.9	**3.9**	**No**	**No**	**No**	**Alive, post-operative f/u**
Stokes MB et al., 2015 (8)	47, male	HVAD	13 months	48 months	Severe pHTN	Yes	Yes - 2 months	47	34	3.2	**1.7**	**NR**	**NR**	**NR**	**Alive, 1-month f/u**
Dakkak AR et al., 2014 (9)	41, female	Heart Mate II	11 months	72 months	Cardiogenic shock	Yes	NR	68	NR	3.1	**NR**	**NR**	**NR**	**NR**	**Alive, post-operative f/u**
George RS et al., 2007 (10)	17, male	Heart Mate I	13 months	36 months	Cardiogenic shock	No	–	–	–	–	**-**	**NR**	**NR**	**NR**	**Alive, 4.5-year f/u**
Arendt K et al., 2010 (11)	28, male	Heart Mate II	21 months	NR	Cardiogenic shock	Yes	Yes - 11 months	NR	NR	16.5	**2.7**	**NR**	**NR**	**Yes**	**Alive, 3-week f/u**
Gyoten T et al., 2020 (12)	37, female	HVAD	30 months	NR	Severe pHTN	Yes	Yes - 30 months	54	20	6.9	**2.8**	**No**	**No**	**Yes**	**Alive, 8-year f/u**
	37, female	HVAD	7 months	NR	Severe pHTN	Yes	Yes - 7 months	55	25	4.5	**2.3**	**Yes**	**No**	**No**	**Died 3 days after OHT of multiple organ failure**

*VAD, ventricular assist device; NR, not recorded; PAP, Pulmonary Artery Pressure; PVR, pulmonary vascular resistance; BTT, bridge to transplantation; pHTN, pulmonary hypertension; OHT, orthotopic heart transplant, f/u: follow up*.

The average age of the patients at VAD implantation was 33 ± 9 years of age. 9 patients were male and four were female. The most common indication for VAD implantation was cardiogenic shock, noted in 5 cases. Heart mate II was implanted in 7, HVAD in 4 and Heart mate I in two of them. The average time on VAD prior to heart transplant was 17 ± 17 months. Drive line infection was reported in 5 patients, of which one required device exchange (5). One patient had a minor (non-disabling) stroke with complete recovery. He was ultimately transplanted but died 3 days after heart transplant of multiorgan failure. No pump thrombosis was noted prior to heart transplant.

Periodic echocardiograms were obtained to adjust the speed of each device. In general, the common speed range is 8600–9000 RPM for HMII, 2200–2800 for the HVAD, and 5200–5800 RPM for the HM3 (5). Our patient's RV chamber size decreased significantly after increasing the speed from 2740 to 2780 rpms.

Pulmonary hypertension (PHT) is a complication that is increasingly seen (3–18%) in this population (2). PHT may be prohibitive for heart transplantation since a PVR >3 Woods Units (WU) and transpulmonary gradient (TPG) >15 mmHg is associated with increased risk of mortality due to failure of the normal donor pulmonary ventricle after transplantation (13). VAD can be used in these cases as a bridge-to-decision and help decrease the pulmonary pressures over the course of several months, so that heart transplant can be possible. To our knowledge, this is the second report of complete normalization of PVR following VAD implantation into a systemic RV in <4 months (8). Data on pulmonary pressures was available on 11 patients prior to VAD implant and results are summarized in Table 2. Only 5 patients had pre implant and post implant pulmonary pressure data. The average of the mean PA pressure before VAD implant was 58 ± 10 mmHg (*n* = 5) and 30 ± 9 mmHg (*n* = 5) after implant (two-tailed *P*-value is <0.0001 using paired *t*-test). The median of the PA pressure was 55 ± (50, 67) mmHg before and 30 ± (22, 39) mmHg after VAD implant. 4 out of 6 patients decreased their PVR to <3 WU after VAD support. None of the five patients reported by Maly et al. (3) had PVR > 3 WU before VAD implantation. In the case reported by Stokes et al. (8), PVR improved from 3.2WU pre-VAD to 1.7WU in 8 weeks. The adult patient reported by Stewart et al. (6) had PVR of 8WU before the VAD and was transplanted 8 months later, but PVR on VAD support is not reported. Three of the seven patients, reported by Michel et al. (7), had PVR > 3 WU, two of them in supra-systemic range. In one of them, PVR decreased from 8.9 to 3.9 WU after 8 months of support. Gyoten T et al. (12) reported two patients with pre-VAD PVRs of 6.9 and 4.5 WU. They were both transplanted and PVR on VAD support was 2.8 and 2.3 WU at 30 and 7 months, respectively. Other authors describing VADs in patients with Mustard switch did not mention PVR (5). Our patient was supported on VAD for nearly 2 years 10 months prior to his eventual transplantation and he did well-overall without any major complications.

In general, patients with CHD have higher perioperative and one-year mortality after heart transplant. However, Pigula et al. showed that the results of heart transplantation in ACHD were comparable to those of adults without a CHD (14). Among the case reports reviewed here, only one patient died 3 days after heart transplant of multiorgan failure. Of the 12 patients who survived the heart transplant, the short-term outcome was good, and one patient was doing well after 8 years.

## Conclusion

As demonstrated, ventricular assist devices may be a safe and viable option to serve as a bridge to transplant/bridge to decision, in Mustard patients with systemic RV failure and secondary pulmonary hypertension. Additionally, we found that VAD therapy can reduce pulmonary pressure and decrease the PVR to normal ranges in a relatively short amount of time. Finally, VADs may serve this group of patients who are not immediately able to be transplanted due to donor supply shortage or hemodynamic instability. In the future, this can possibly be expanded to failing systemic right ventricles in patient with other types of complex congenital heart disease.

## Data Availability Statement

The original contributions presented in the study are included in the article/supplementary material, further inquiries can be directed to the corresponding authors.

## Ethics Statement

Ethical review and approval was not required for the study on human participants in accordance with the local legislation and institutional requirements. The patients/participants provided their written informed consent to participate in this study.

## Author Contributions

RB took the lead in writing the manuscript. All authors cared for the patient, provided critical feedback, and helped shape and edit the final manuscript.

## Conflict of Interest

The authors declare that the research was conducted in the absence of any commercial or financial relationships that could be construed as a potential conflict of interest.
